# Development of polylactic acid-based nanomat with silver nitrate and betel leaf extract for antimicrobial food packaging

**DOI:** 10.1039/d6ra03451a

**Published:** 2026-07-06

**Authors:** Md Golam Mortuza Limon, Md Abdus Shahid, Imam Hossain, Azizur Rahman, Selim Reza, Mst. Hurazannat Monira

**Affiliations:** a Department of Textile Engineering, Dhaka University of Engineering and Technology Gazipur 1707 Bangladesh shahid@duet.ac.bd; b Department of Textile Engineering, Northern University Dhaka 1230 Bangladesh; c Department of Microbiology, Primeasia University Banani Dhaka Bangladesh

## Abstract

The growing environmental effects and safety issues associated with the use of traditional petroleum-based packaging materials have increased the need for sustainable and antimicrobial products. In the present work, an electrospun polylactic acid (PLA) nanomat containing silver nitrate and betel leaf (*Piper betle* L.) extract was fabricated for food packaging with antimicrobial properties. The electrospinning method achieved pure PLA and nanomats with different extract concentrations. Scanning electron microscopy (SEM) confirmed that electrospun fibers were uniformly formed, and the fiber diameter as well as porosity decreased with increasing extract loadings, leading to a more compact nanofiber structure. Fourier-transform infrared spectroscopy (FTIR) and energy dispersive X-ray (EDX) examination confirmed the successful presence of silver and bioactive phytochemicals through the PLA matrix. Moisture management testing (MMT) confirmed superior waterproofing of the composite nanomats. Antibacterial and antifungal studies indicated significant inhibition of *Staphylococcus aureus*, *Escherichia coli*, and *Aspergillus niger*, where the nanomat demonstrated the most efficient antimicrobial action owing to the combined effects of betel leaf extract and silver ions. From the thermal analysis, the differential scanning calorimetry (DSC) and thermogravimetric analysis (TGA) of developed nanomats were better than those of pure PLA. Analysis of mechanical performance (tensile and bursting) showed that it has acceptable tensile and bursting strength characteristics for packaging purposes. These results indicated that prepared PLA-based nanomats possessed improved antimicrobial, thermal, and moisture barrier activities as well as mechanical properties, supporting the potential of their application for eco-friendly and sustainable antimicrobial food packaging materials.

## Abstract 2

PLA nanomat containing silver nitrate and betel leaf extract was fabricated through electrospinning, exhibiting moisture resistance, sufficient thermal stability, antibacterial and antifungal activity for food packaging.

## Graphical abstract

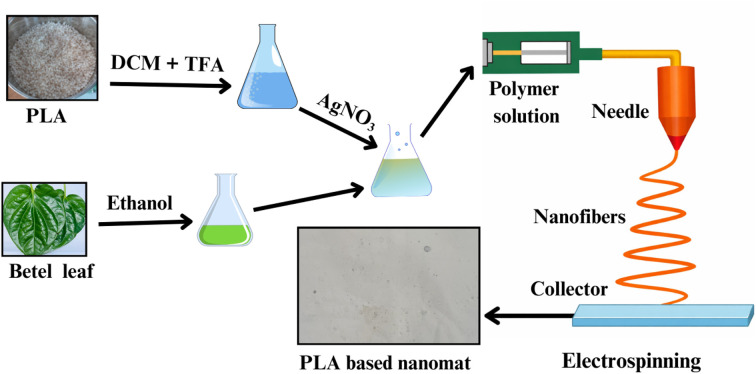

## 1. Introduction

Food packaging plays an important role for maintaining food quality, protecting products from physical damage, limiting contamination, extending shelf life and providing consumers with product information.^1,2^ Most packaging materials are made from petroleum-based plastics, which are harmful for the environment and resistant to natural degradation.^3,4^ As many synthetic polymers do not degrade for long periods of time, plastic packaging waste considerably adds to landfill pollution, marine pollution and environmental contamination.^5–7^ Meanwhile, food loss and waste continue to be severe global challenges in which microbial spoilage is a major contributor to food degradation at later stages of the supply chain, namely during storage, distribution and retail handling. Microorganisms (*e.g.*, bacteria, yeasts and molds) spoil food quality both sensorially and nutritionally; in addition, they negatively affect its safety due to not only the production of toxic chemicals but also the growth of pathogenic microorganisms.^7–10^ Traditional plastic packaging offers a mechanical barrier but lacks active inhibition of microbial proliferation either on the food surface or within the package environment. Accordingly, the drawbacks of traditional packaging are environmental and food safety concerns.^11,12^ The most recent concerns over plastic pollution, food spoilage and food waste have drawn attention to the need for sustainable active packaging systems with biodegradable materials combined with antimicrobial functionality.^13,14^

Biodegradable polymers have aroused a great interest during the last decades to overcome several difficulties related to petroleum-based plastics; for example, PLA as one of the most widely studied and used biodegradable polymer.^15–17^ PLA is a common focus for food packaging as it can be obtained from renewable resources (*e.g.*, corn starch and sugarcane) and has biodegradability, biocompatibility, processability, and less environmental burden compared with conventional plastics.^18,19^ However, it has limitations, including poor moisture resistance and limited antibacterial qualities, which may reduce its usefulness in food preservation.^19^ An alternative approach to address these limitations is exerting antimicrobial agents in PLA-based packaging materials. Active antimicrobial packaging that inhibits or delays microbial growth can prolong the lag phase of spoilage microorganisms to extend shelf-life while reaching their desired quality for a minimum period, leading to enhanced food safety and less spoilage-associated waste generation.^20–22^

Betel leaf is a natural herbal leaf and has been reported to have antibacterial, antifungal, and antioxidant properties.^23,24^ Plant extracts, such as betel leaf, have been demonstrated in studies to improve the antibacterial qualities of packing materials.^23–25^ Some studies have been done by the solution casting method for creating PLA-betel leaf nanomats.^26^ These works lacked the enhanced surface area and porosity of the product, limiting active agent efficiency and synergistic antibacterial effects.

In this case, electrospun PLA nanomats incorporated with betel leaf extract has been previously investigated for food packaging applications.^27^ However, previous work was limited by comprehensive characterization and there has been little investigation into extract concentration or the long-term antimicrobial performance. Thus, an in-depth analysis on multiple PLA nanomat formulations loaded with varity of betel leaf extract is required. This study focuses on electrospun PLA nanomats and in the incorporation of betel leaf extract and silver nitrate in the creation of active food packaging. Electrospinning was chosen as it has the potential to create high-surface-area-to-volume nanofibrous mats, interconnected porosity with active compounds exposed and thus facilitating good antimicrobial packaging performance.^27,28^

Silver nitrate (AgNO_3_), known as an inorganic antimicrobial agent due to the release of silver ions (Ag^+^), was added since Ag^+^ have been reported as effective at inhibiting bacterial growth through a combination of mechanisms.^29^ Ag^+^ can also bind to thiol groups, interfering with metal-dependent enzymes and resulting in enzyme inhibition related to disturbed respiration and metabolic activity. It can also disrupt bacterial membrane, interact with nucleic acids, and promote the generation of reactive oxygen species leading to oxidative stress as well as cellular damage.^30,31^ AgNO_3_ can exhibit complementary antibacterial effects when combining with bio-active phytochemicals found in betel leaf extract possessing functionality as antimicrobial and antioxidant, hence contributing to functional performance improvements of PLA nanomats.^32,33^

Therefore, current works focusing on electrospun PLA-based nanomats containing betel leaf extract and silver nitrate with potential application as active food packaging materials. The research aims at providing enhanced antibacterial activity and moisture-control characteristics, in conjunction with being a more sustainable replacement for traditional fossil fuel-based plastic packaging.

## 2 Materials and methods

### 2.1 Materials and chemicals

PLA was sourced from China, which is 100% biodegradable. Trifluoracetic acid (TFA) and dichloromethane (DCM) were collected from Hatkhola, Dhaka, Bangladesh. Distilled water, ethanol and betel leaves were procured from the local market, Gazipur, Bangladesh. DOW Chemical International Pvt. Ltd in India was the source of Silvadur 930 FLEX, an antibiotic that contains silver nitrate (concentration ≥0.1–<0.25%), ammonia (concentration ≥0.25–<1.0%), and ethanol (concentration ≥1.0–<10.0%). Silvadur 930 FLEX a silver-based antimicrobial additive was utilized. The silvadur results in an aqueous-based silver-polymer delivery system that sequesters and releases low levels of silver ions.

### 2.2 Formulation of PLA polymer solution

Pellets of PLA were dissolved in 20% (w/v) solution to achieve a grade suitable for spinning. Binary solvent system, DCM and TFA, 80% : 20% by volume, were used. The polymer-solvent blend was allowed to stir at room temperature for 24 h to ensure complete solubility. The mixture of a total of 400 mL obtained was magnetically stirred for 60 min to form a uniform and stable PLA solution.

### 2.3 Extraction of betel leaf constituent

Fresh betel leaf was thoroughly washed with pure water to get rid of the surface impurities. Leaves were then slashed into small pieces and then sun-dried, eliminating the moisture. The dried leaves were powdered and used for the study. The powder of 20 gm was contained in a bottle sealed after being filled with 300 mL of ethanol, and stored at room temperature for 48 h to elute out the solvent. The ethanolic extract was kept for subsequent use.

### 2.4 Preparation of electrospinning feed solution

To investigate the effect of betel leaf extract and silvadur on the physical, mechanical, surface and thermal properties of PLA nanomat, a total of four electrospinning feed solutions was prepared. Control sample contained a pure PLA solution. Further formulations were prepared by adding varying volumes of the ethanolic betel leaf extract and silvadur to the PLA solution (Table 1). Mixtures were stirred prior to electrospinning to achieve better dispersion of the active constituents within the PLA solution. Additionally, PS and PB samples were prepared with same electrospinning condition for the assessment of antibacterial performance of silvadur and betel leaf respectively.

**Table 1:** Experimental design and composition of PLA-betel leaf-silvadur composite samples

Sample no.	Sample code	PLA content (mL)	Betel leaf extract (mL)	Silvadur 930 FLEX (mL)	Description
1	PPM	30	—	—	Pure PLA control
2	PBS-1	27	3	1	PLA with low betel leaf extract and silvadur
3	PBS-2	24	6	1	PLA with low betel leaf extract and silvadur
4	PBS-3	21	9	1	PLA with low betel leaf extract and silvadur

### 2.5 Fabrication of PLA nanofibrous mat

Nanofiber fabrication was conducted in a single electrospinning set-up containing a high-voltage power supply, a collector of a rotating drum (diameter of 158 mm, length of 500 mm) at the rate of 500 rpm, a syringe with volume ranged from 20 mL and heating module model (0.50 kW; Tong Li Tech-meanwhile, needles with gauge size-20; TL-Pro-BM: Manufacturer; China). The resultant solutions were transferred to a pump container (the pump container has a capacity of 30 mL) through the needle set. Spinning conditions were optimized experimentally. The optimal parameters were a voltage of −10.5 kV (collector) and +20.5 kV (needle), solution flow rate of 2.0 mL h^−1^, and ambient temperature and humidity of 27 °C and 65%. The nanofiber mats obtained after regeneration were collected on aluminum foil, dried overnight, and conditioned before characterization.

## 3 Characterization

### 3.1 Physical property

#### 3.1.1 Scanning electron microscopy (SEM)

The morphological nature of the samples was observed by SEM (Hitachi SU-1510, Japan) to take high-quality images at magnifications of 500 00×. The imaging was done using a 5 kV voltage. ImageJ software was used for precision analysis to measure diameter distributions within the sample.

#### 3.1.2 Energy dispersive X-ray spectroscopy (EDX)

EDX spectroscopy analysis using a field emission scanning electron microscope (FE-SEM, JSM 7610F, JEOL, Japan) at an energy range of 0 to 20 keV was also performed. It is used to determine the mass% of different elements present in the samples.

#### 3.1.3 Fourier-transform infrared spectroscopy (FTIR)

FTIR; IRPrestige21, Shimadzu Corporation, Japan, was used to analyze the chemical structure of the samples. Spectra were collected between 500–4000 cm^−1^, at a rate of 4 cm^−1^ to obtain the functional groups and molecular nature of the material.

#### 3.1.4 Moisture management test (MMT)

Moisture management properties of the samples were characterized on a Moisture Management Tester (M290, SDL Atlas, UK) according to the AATCC 195-2009 standard practice. Some moisture interaction parameters, such as wetting time, absorption rate, maximum wetted radius, and spreading speed on both inner and outer surfaces, were evaluated under the test. The resulting total one-direction of transport capacity (*R*) and overall moisture management capacity (OMMC) were then calculated. It was with saline solution (0.9% sodium chloride) and a 120 second reading time for assessment of the moisture behavior of the nanomat samples.

### 3.2 Mechanical property

#### 3.2.1 Tensile strength

The tensile characteristics of the PLA-based nanomats were evaluated using a James Heal tensile testing machine for their resistance to uniaxial tensile force. The measurements were performed according to standard EN ISO 13934-2 (grab method), which measures the maximum force and elongation until breakage of the nanomat. The specimens were stretched under an increasing load, and the load (N) or elongation (%) applied was continuously monitored until failure. Maximum force (N), elongation at maximum force (mm), and time to fracture (s) were derived from the force–elongation curves.

#### 3.2.2 Bursting strength

The resistance of the developed PLA-based nanomats to multidirectional pressure was assessed using a TruBurst^4^ bursting strength tester (James Heal, UK) following the ISO 13938-2:1999 standard by use of the diaphragm method. The samples were fixed, and hydraulic pressure was applied in a controlled manner until breakage. From each specimen, bursting pressure (kPa), bursting extension (mm), and the time to burst (s) were noted.

### 3.3 Thermal property

To assess the thermal stability, degradation behavior, and thermal transitions of the samples, we used a Simultaneous Thermal Analyzer (STA 449 F5 Jupiter, Germany) that integrates TGA, DTG, and DSC. The testing ranged from ambient temperature to 600 °C to discover the material's thermal features.

### 3.4 Biological property

#### 3.4.1 Antibacterial test

The antibacterial activity of the nanomat samples were assessed through the disc diffusion method against *S. aureus* (ATCC 25923) and *E. coli* (ATCC 25922).^34^ The bacterial cultures were maintained on nutrient agar (NA; 0.5% peptone, 0.3% beef extract, 1.5% agar, agar 15 gm per thousand mL, pH: 7) plates. We used 24 h fresh culture on nutrient agar plate for this experiment. All bacterial cultures were picked (1 loop of) into 9 mL of freshly prepared normal saline where 8.5 gm of NaCl was taken to 1000 mL of distilled water. Then incubated in 37 °C temperature for 30 min. After incubation all test tube are shaking speed 150 rpm for approximately 10 min beforehand use in experiments. The optical density (600 nm) of the bacterial suspension was adjusted to 0.5 McFarland standard (∼1.5 × 10^8^ CFU mL^−1^).

Bacterial suspensions were spread uniformly on the surface of Muller Hinton agar (MHA; 2.0% beef extract, 1.5% casein hydrolysate, 1.5% agar, pH 7.2) plates with a sterile cotton swab in a single direction for use as an inoculum for the bioassay against *P.cactus* and *S.apiospermum* strains in order to have uniform distribution of bacteria on each plate. Nanomat samples and antibiotic standard control discs were aseptically placed on the agar surface with sterile tweezers. The plates were incubated at 37 °C for 24 h and the antibacterial activity was ascertained by measuring the zone of inhibition (ZOI) that appeared around each sample on a blue background. All assays, both technical and biological replicates were performed in triplicate.

#### 3.4.2 Antifungal test

The antifungal activity of the nanomats were evaluated against *Aspergillus niger* (ATCC 6275) disc diffusion assay.^35^ Fungal cultures were maintained on potato dextrose agar (PDA; 2% dextrose, 1.5% agar, pH = 5.6) plates in the dark. A loopful of 7 day old fungal culture grown in PDA was inoculated in PDA broth and incubated at 25 °C until turbidity attained equivalent to 0.5 McFarland standard.

The fungal suspension was streaked evenly across the surface of PDA plates with a sterile cotton swab. Using sterile forceps, nanomat samples and control discs were placed aseptically onto the plates inoculated with the various strains. Plates were incubated at 25 °C for 48 h in room temperature and then the ZOI around each sample was measured in mm. All assays were performed in triplicate with technical and biological replicates.

## 4 Result and discussions

### 4.1 Developed sample

**Fig. 1:**
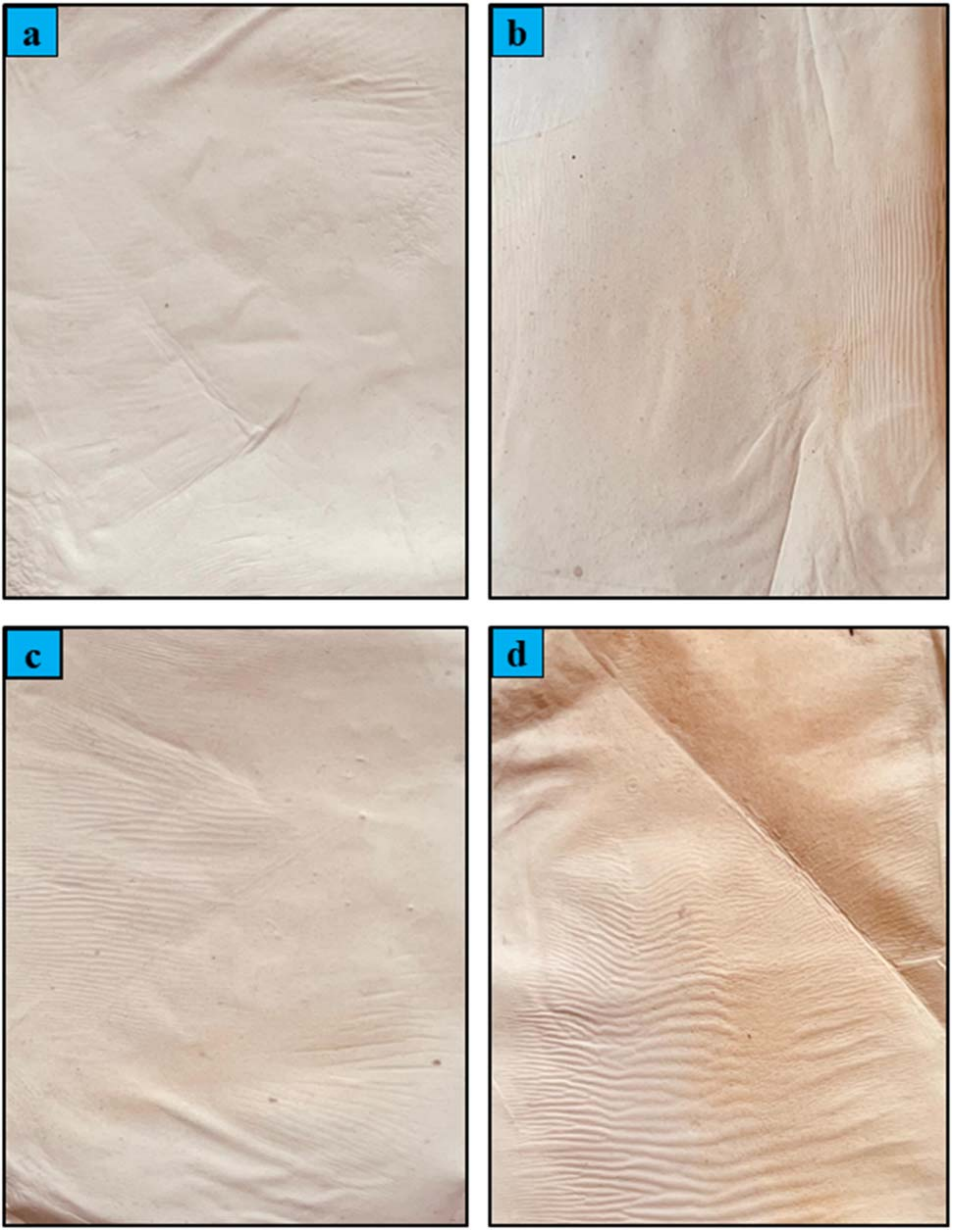
Developed samples of (a) PPM, (b) PBS-1, (c) PBS-2, and (d) PBS-3.

During the electrospinning, three different samples were fabricated by changing the concentration of betel leaf (%w/v) in electrospinning solution with pure PLA mat. Fig. 1 shows the three nanomaterial samples plotted alongside the PPM sample.

**Fig. 2:**
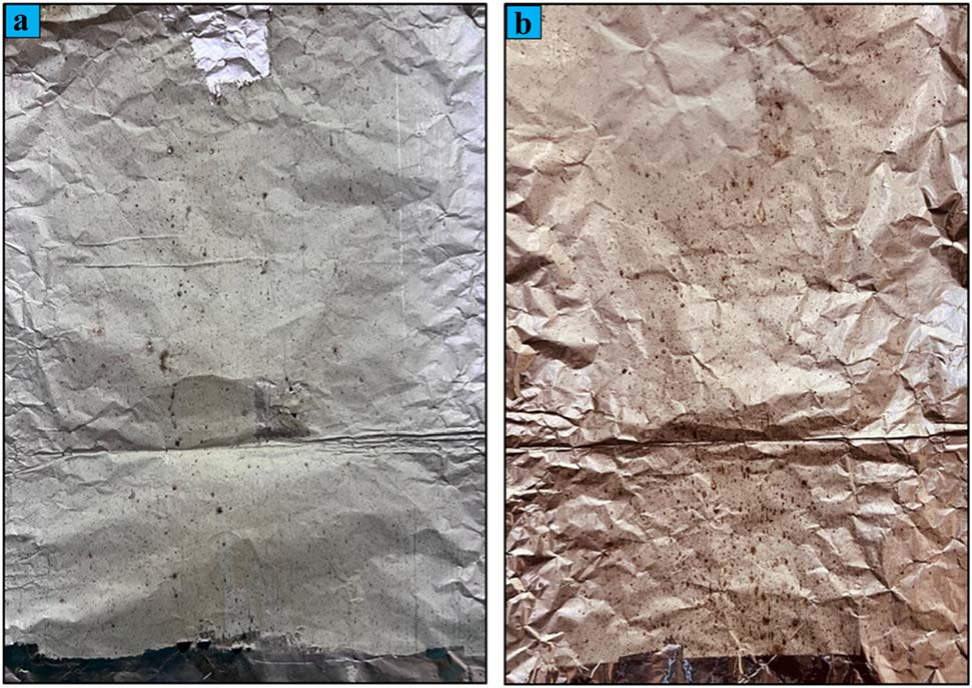
Developed samples using (a) 35% betel leaf and (b) 40% betel leaf concentration.

Betel leaf samples with concentration of 35% and 40% could not be processed as the electrospun solution did not have enough viscosity and resulting nanomat was too brittle, respectively, as shown in Fig. 2.

### 4.2 Morphological analysis

#### 4.2.1 SEM analysis

The morphological, diameter distribution and porosity characteristics that are of direct relevance to potential antimicrobial food packaging properties were assessed using SEM on the electrospun nanomats. The four samples were PPM, PBS-1, PBS-2, and PBS-3 which each underwent analysis.

**Fig. 3:**
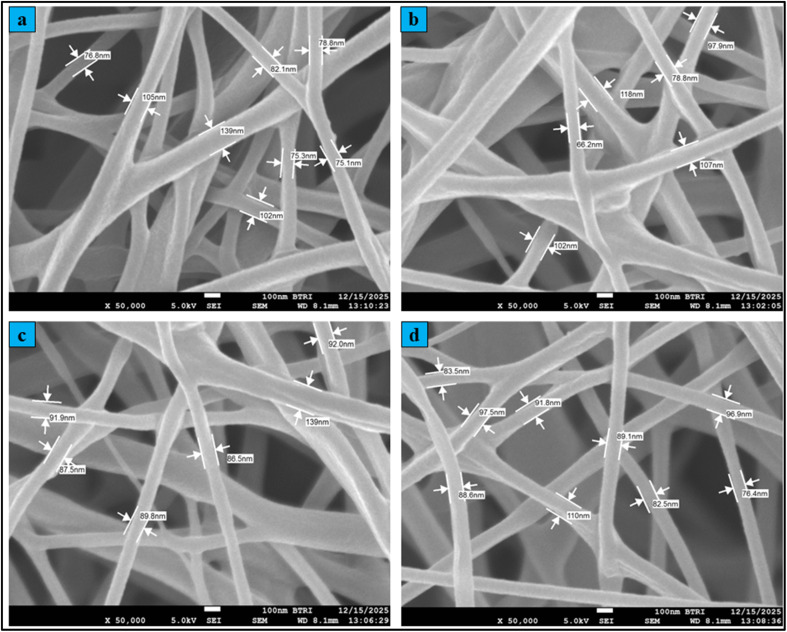
SEM image of (a) PPM, (b) PBS-1, (c) PBS-2, and (d) PBS-3.

**Fig. 4:**
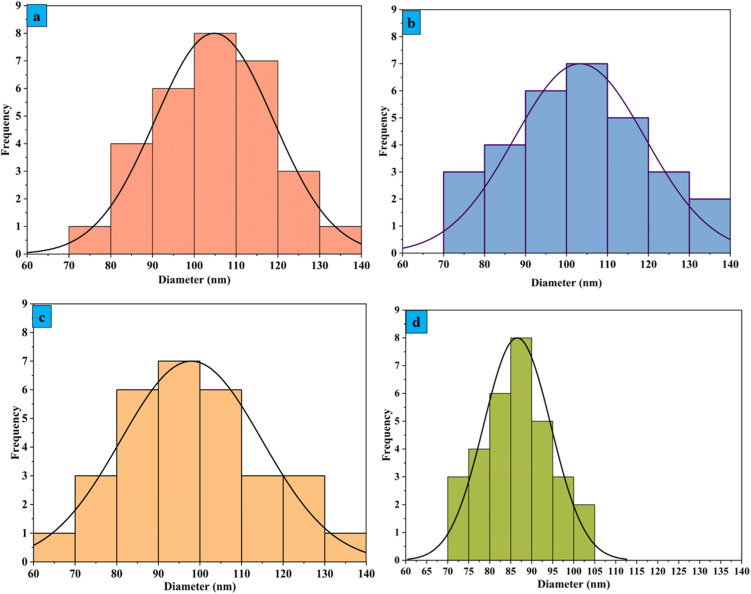
Diameter distribution analysis of (a) PPM, (b) PBS-1, (c) PBS-2, and (d) PBS-3.

The SEM images of PPM have smooth and even superfine fibers with free beads without defects, as shown in Fig. 3. The average fiber diameter of PPM was 104.76 ± 13.75 nm. PBS-1 fibers retained continuity and uniformity with an average diameter of 103.27 ± 16.00 nm. PBS-2 fibers showed slightly reduced uniformity, averaging 97.94 ± 16.96 nm. PBS-3 exhibited the thinnest fibers (86.55 ± 8.02 nm), forming a denser, more closely packed network shown in Fig. 4. Reduced fiber diameter may be due to a higher extract content and lower PLA concentration that together stimulate the conductivity of the solution, thus providing greater stretching of the jet during electrospinning.^36^

**Fig. 5:**
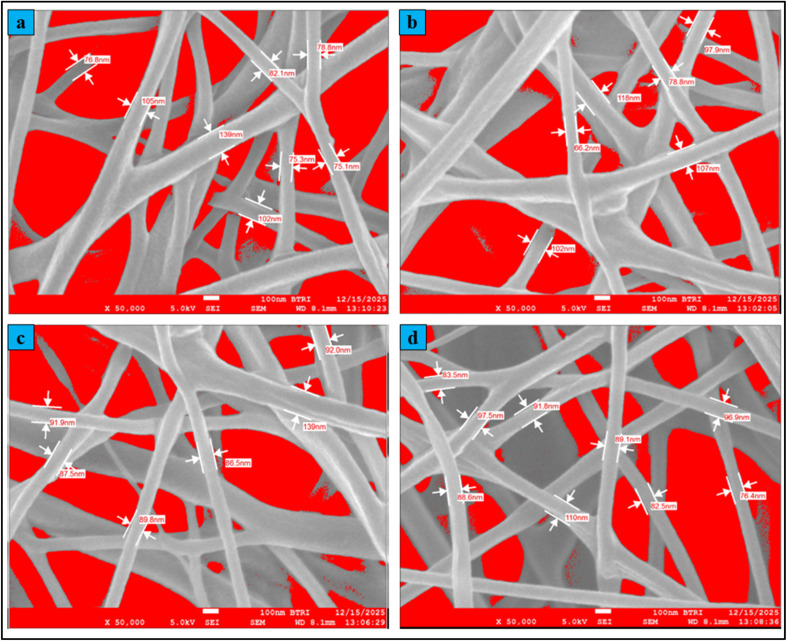
Porosity analysis image of (a) PPM, (b) PBS-1, (c) PBS-2, and (d) PBS-3.

The porosity analysis (Fig. 5) showed that the extract concentration was inversely proportional to pore area: 39.08% (PPM), 35.12% (PBS-1), 32.43% (PBS-2), and 28.63% (PBS-3) from SEM porosity images. Although thin fibers and tighter packing reduce porosity, variability in % PLA and % extract between samples was shown to affect fiber diameter and porosity as well.^37^

#### 4.2.2 Surface roughness analysis

**Fig. 6:**
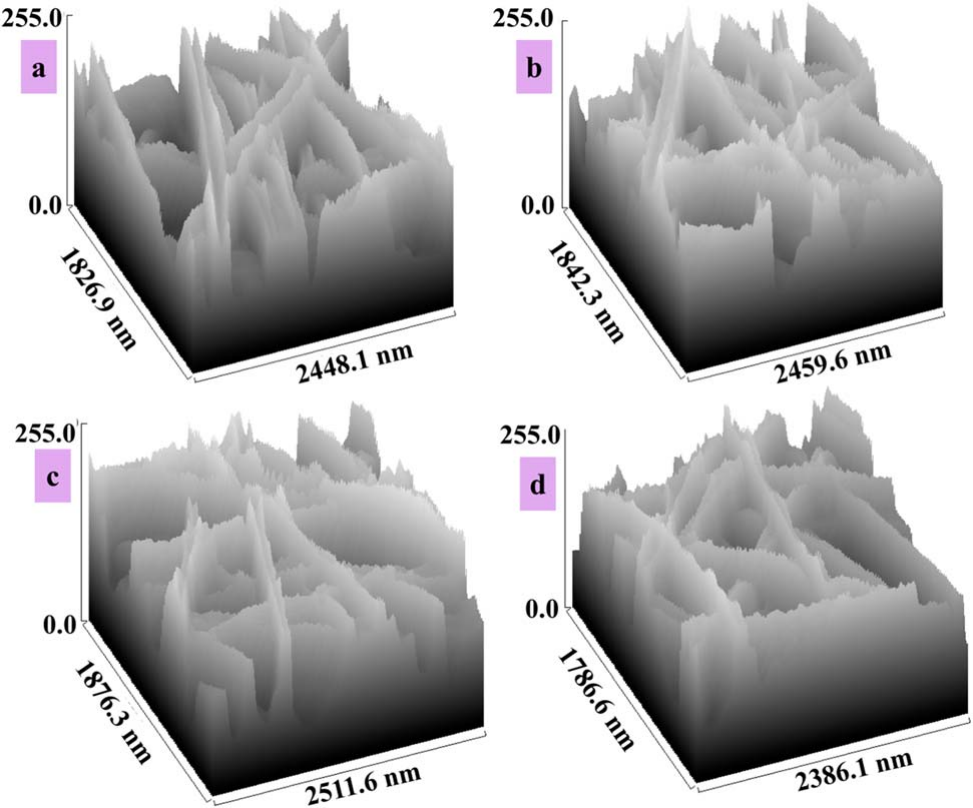
3D surface roughness plot using imageJ of the developed samples (a) PPM, (b) PBS-1, (c) PBS-2, and (d) PBS-3.

3D surface topography of nanomats (PPM, PBS-1, PBS-2, and PBS-3) based on their SEM images analyzed by ImageJ, is shown in Fig. 6(a–d). This analysis was conducted to see how the surface roughness varies as the betel leaf extract and silver nitrate concentration are augmented in the PLA matrix.

As in Fig. 6(a), the PPM sample possesses a relatively smooth and flat surface having fine nano-scale protrusions. This less rugged topology is characteristic of pure PLA, where uniform electrospinning results in a homogeneous fiber layer and barely has non-uniformity.

The PBS-1, PBS-2 and PBS-3 samples are shown in Fig. 6(b–d) by increasingly rougher and larger peak-valley features. Increased extract and Silvadur concentrations yield higher solution conductivity, resulting in uneven fiber stacking and pointed surface features. With the formation of larger and sparser peaks, a more complex topography is created, which reduces the direct area of contact with microorganisms; however, these peaks remain able to concentrate the bioactive agents locally and may also promote an increase in local interactions between them at specific points on the surface which enhances antimicrobial effectiveness.

#### 4.2.3 EDX analysis

**Fig. 7:**
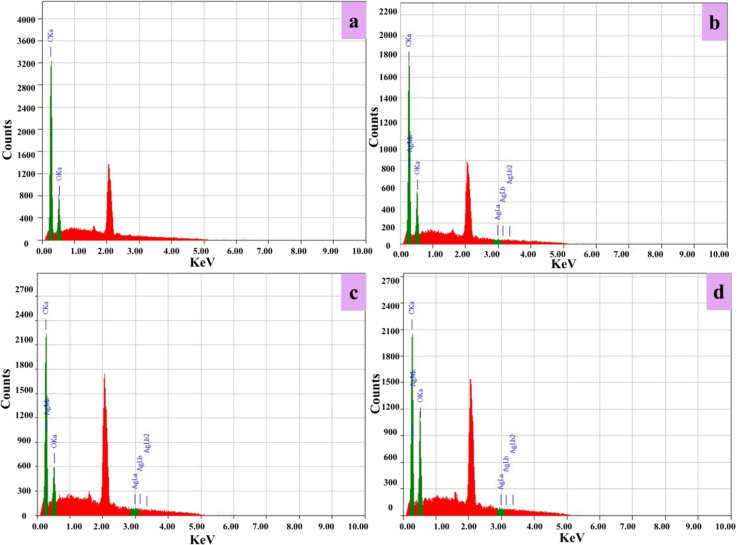
EDX spectra of (a) PPM, (b) PBS-1, (c) PBS-2, and (d) PBS-3.

EDX analysis of the samples demonstrated in Fig. 7 that indicates the elemental compacts were significantly different as content for betel leaf extract and silvadur nanoparticles increased in PLA matrix. Regarding to the PPM-100% PLA nanomat, it included 72.89 mass% of carbon and 27.11 mass% of oxygen with no Ag was detected, demonstrating its nature is a pure PLA material. In PBS-1, carbon content reduced to 68.93%, while oxygen rose to 27.19%, also trace element of silver (3.88%) was observed and could be attributed to addition of silvadur nanoparticles. PBS-2 reported a lower carbon content of 50.02%, higher oxygen content of 43.39% but significant increase in silver content up to 6.59%. At last, PBS-3 was the candidate with high content of functional agents and had carbon-63.18%, oxygen-28.10%, silver-8.72%. The slight decrease of carbon probably indicates the reduced PLA amount in the PBS samples, while the enhanced oxygen content denotes incorporation of oxygenated betel leaf extract. The detection of silver also proves the existence of silvadur nanoparticles. Because the silvadur content in all PBS samples was constant, the difference in measured silver percentage likely come from either local distribution or a measurement variation when performing EDX rather than varying betel leaf extract content.

### 4.3 Chemical analysis

#### 4.3.1 FTIR analysis

**Fig. 8:**
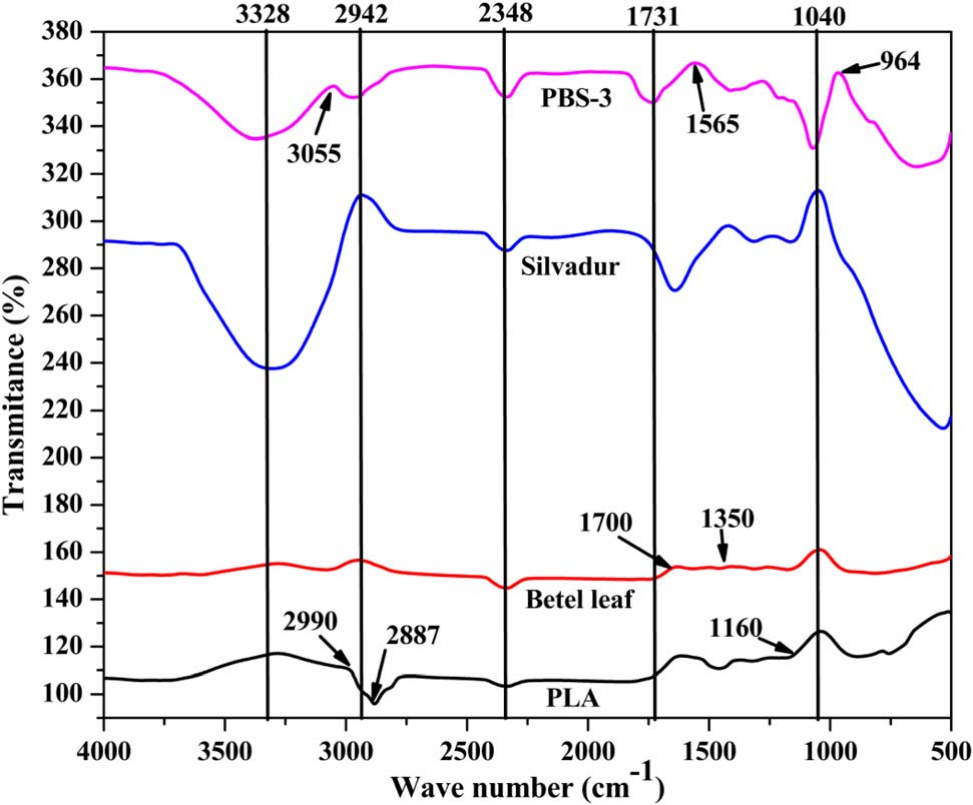
FTIR analysis of PLA, betel leaf, silvadur, and PBS-3 samples.

FTIR spectroscopy was performed to characterize the functional groups present in each component and ensure that all materials were integrated into the fabricated nanomat shown in Fig. 8. PLA spectrum displays its characteristic peaks at 2990–2887 cm^−1^ (C–H stretching), 1755 cm^−1^ (C=O ester) and 1160 cm^−1^ (C–O–C), confirming the classic polyester backbone.^27^ Betel leaf extract showed peaks at 3328 cm^−1^ (O–H stretching of phenolics), 2942 cm^−1^ (C–H stretching), 1350 and 1040 cm^−1^ (aromatic O–H bending, C–O stretching) revealing the existence of bioactive phytochemicals.^27^ Characteristic absorptions of Ag-based complexes and ligands are observed at approximately 2942, 2348, 1731 and 1040 cm^−1^ on the FTIR spectrum of silvadur.^38,39^ For the combined nanomat (PBS-3), the overlap and slightly shift in the peaks such as PLA ester band at 1731 cm^−1^, enhanced O–H stretching mode of 3328 cm^−1^ due to betel extract and silver-complex vibrational bands located near 1565 cm^−1^ and 1040–964 cm^−1^ suggest that betel leaf extract along with silvadur had been successfully included within PLA matrix.

### 4.4 Moisture management analysis

The moisture absorption, retention, and transport properties of the four samples, for food packaging applications, were assessed using the moisture management test. The test evaluates wetting time, absorption rate, spreadability, and one-way transport capability (OWTC) to determine how well the materials control moisture, a key factor in keeping food fresh and high-quality.

**Fig. 9:**
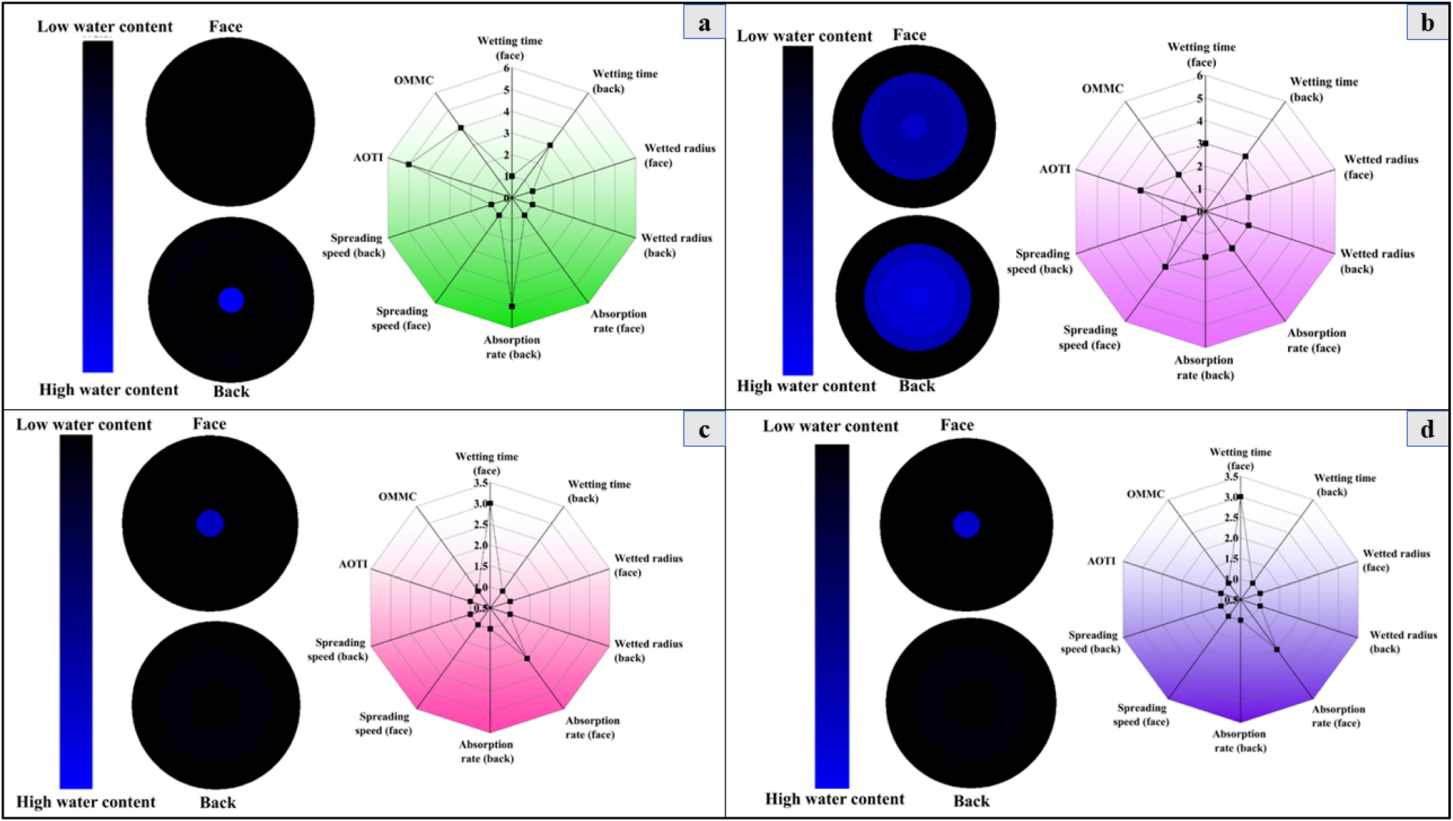
Water location *vs.* time diagram and radar diagram of (a) PPM, (b) PBS-1, (c) PBS-2, and (d) PBS-3.

The OWTC of the PPM sample was about 862.21, which was assigned as high for moisture transport property, indicating the PPM sample is made up of 100% PLA nanofiber, as shown in Fig. 9(a).^17^ However, it did not present any absorption or spreading because the PLA is hydrophobic. PBS-1 sample depicted the most reduction for OWTC about 127.0156, increased absorption rate of 9.1139%/s, and wetting time of 10.109 s, which means enhancing water holding capacity but decreasing transportation capability. PBS-2 presented a negative OWTC value equal to −110.84. The sample was wet but did not effectively “wick” the water, indicating the waterproofing effect. PBS-3 sample exhibited higher water resistance (*i.e.*, more negative value of OWTC: −124.681) than the other ones, and also the best absorption, and the slowest spreading speed.

The inclusion of silvadur, for the last three samples PBS-1-PBS-2 and PBS-3, highlighted in Fig. 9(b–d), likely added to the anti-microbial properties observed; however, it may also be attributable to the moisture management properties. Silvadur is, in fact, known to give antimicrobial activity and so would be useful in food packaging for maintaining shelf-life and to prevent microbial contamination of the packaged food. But possibly its presence would also have decreased the moisture transport of the material, as shown by the decreasing OWTC values with a higher concentration of betel leaf extracts. The Betel leaf extract concentration seems to improve the water resistance of the nanomat, possibly by creating a moisture barrier, and the possible band-gap character of silvadur may induce limitation in spreading and transmission, or capture and hold mechanism.

The results obtained imply a trade-off between moisture conduction and water resistance. The 100% PLA film is suitable for moisture diffusion, needing the same conditions as food that uses active packaging application for breathability, and the presence of betel leaf extract at higher levels (with silvadur) yielded in water-resistant films suited better for products demanding protection against external moisture, such as frozen foods or certain liquid-based products. But with the growing water blocking capability, the packaging of products that have to be somewhat regulated, such as, for example, fruits or bakery goods, finds its limits, when moisture exchange is necessary to prevent spoilage.

### 4.5 Biological property

#### 4.5.1 Antibacterial performance analysis

The developed nanomats were assessed for their antibacterial activity by the ZOI method with *S. aureus*, a Gram-positive bacterium, and *E. coli*, a Gram-negative bacterium for potential food packaging and antiseptic applications. All assays were performed in triplicate and results were presented as mean ± standard deviation. The antibacterial activity of the prepared nanomats against both bacterial strains is summarized in Table 2.

**Table 2:** Comparative antibacterial performance of samples against Gram-positive and Gram-negative bacterial

Sample code	*S. aureus* (mm)	*E. coli* (mm)
PPM	0	0
PS	17 ± 2	21 ± 2
PB	19 ± 2	23 ± 2
PBS-3	22 ± 2	26 ± 2

**Fig. 10:**
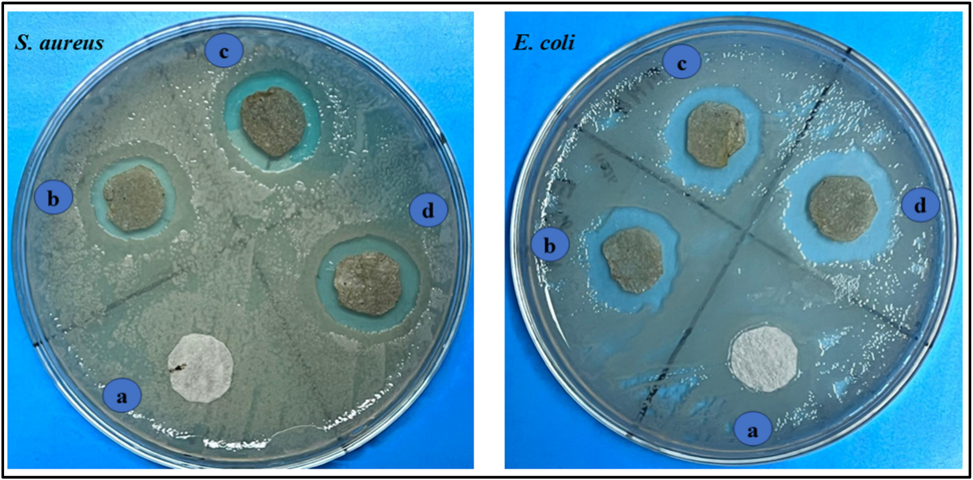
Antibacterial performance against *S. aureus* and *E. coli* using the ZOI method: (a) PPM (pure PLA), (b) PS (PLA + silvadur), (c) PB (PLA + betel leaf extract), and (d) PBS-3 (PLA + silvadur + betel leaf extract).

PPM sample has no ZOI against *S. aureus* and *E. coli* in mm (Fig. 10(a)). This was an expected finding in our study given that pure PLA is a normal polymer that has no inherent antimicrobial ability.^20^ PS sample exhibited excellent antibacterial activities against *S. aureus* (17 ± 2 mm, ZOIs) and *E. coli* (21 ± 2 mm, ZOIs), as shown in Fig. 10(b). Most of the antibacterial activity can be attributed to the realese of silver ions from silvadur; these ions disrupt bacterial cell membranes, block enzymatic activity and inhibit DNA replication.^40^Fig. 10(c) shows PB sample which displayed an increased antibacterial action with ZOIs of 19 ± 2 mm against *S. aureus* and ZOIs of 23 ± 2 mm against *E. coli*. The antibacterial effect may be linked to the bioactive phytochemicals like phenolics, flavonoids, tannins, and essential oils present in betel leaf extract which can kill bacteria by damaging their cell wall and inducing oxidative stress.^27^

The ZOI of PBS-3 (22 ± 2 mm for *S. aureus*, 26 ± 2 mm for *E. coli*) was also significantly higher than both PS (17 ± 2 mm, 21 ± 2 mm) and PB (19 ± 2 mm,23 ± 2 mm) (*p* < 0.05), confirming the increase in antibacterial activity by PBS-3 (Fig. 10(d)). This synergistic mechanism accounts for the increased antimicrobial action, as compounds with broad-spectrum bioactivity present in betel leaf extract act additionally to sustain bactericidal activity from released silver ions of silvadur mixture and result maximum inhibition.

#### 4.5.2 Antifungal assay analysis

**Fig. 11:**
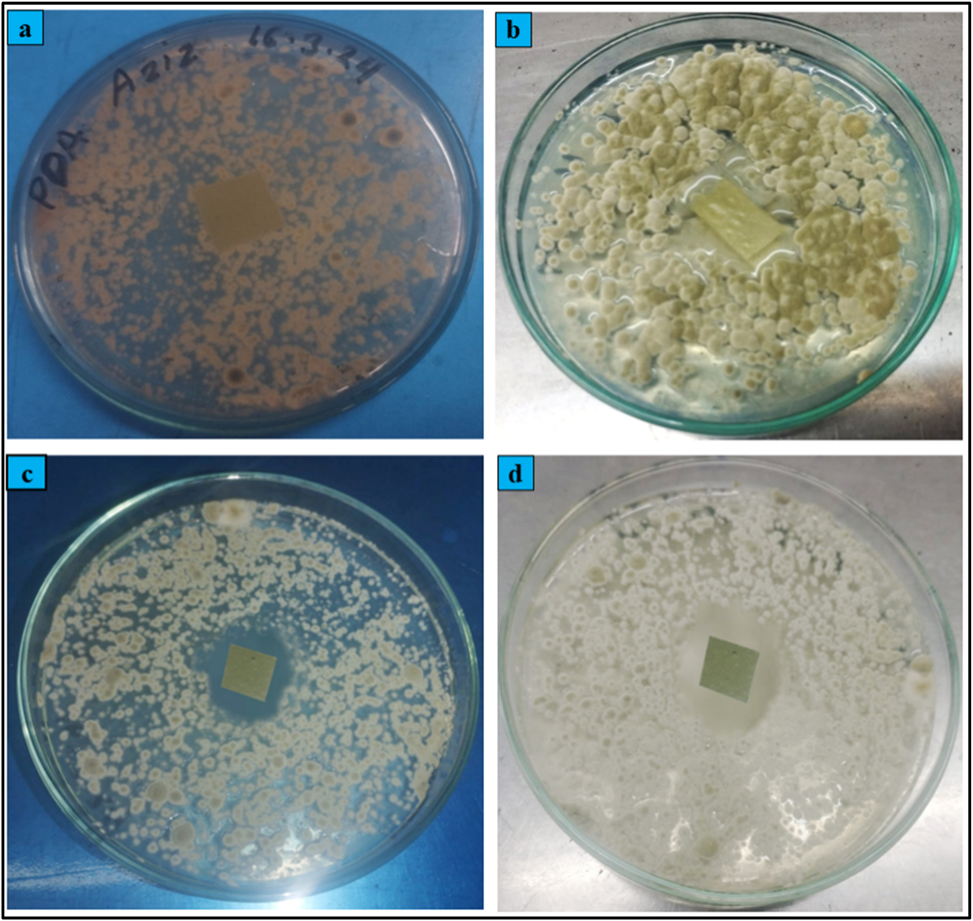
Antifungal performance of (a) PPM, (b) PS, (c) PB, and (d) PBS-3 samples.

Fungistatic activity was determined by the ZOI method to evaluate the inhibition capacity of the nanomats as inhibitors of fungal growth. The PPM sample showed no antifungal activity showed in Fig. 11(a), meaning 0 mm ZOI, as expected, while it is an inert material. The ZOI of silvadur (PS) was 22 ± 2 mm, corresponding to good antifungal inhibition highlighted in Fig. 11(b). The antimicrobial action of silvadur might be owing to the release of silver ions that exert an antifungal effect.^41^ The positive control group of the PB sample showed the highest ZOI equal to 27 ± 2 mm, as represented in Fig. 11(c). This is possible because the presence of antifungal compounds found in the leaves extract, *i.e.*, phenolics and essential oils, affects the surface membrane damage and fungal mycelium germination processes.^27^

PBS-3 showed maximum antifungal activity with 29 ± 2 mm of ZOI observed in Fig. 11(d). The enhanced activity is attributed to the combined fungicidal action against the exclusively lactate-producing fermentative yeasts, where silver ions cause instantaneous cell disruption and bioactive components from betel leaf extract exert long-term antifungal inhibition. This doubly-acting mechanism results in optimal inhibition of the fungal development process, which renders PBS-3 as the best formulation.

### 4.6 Thermal analysis

#### 4.6.1 TG analysis

**Fig. 12:**
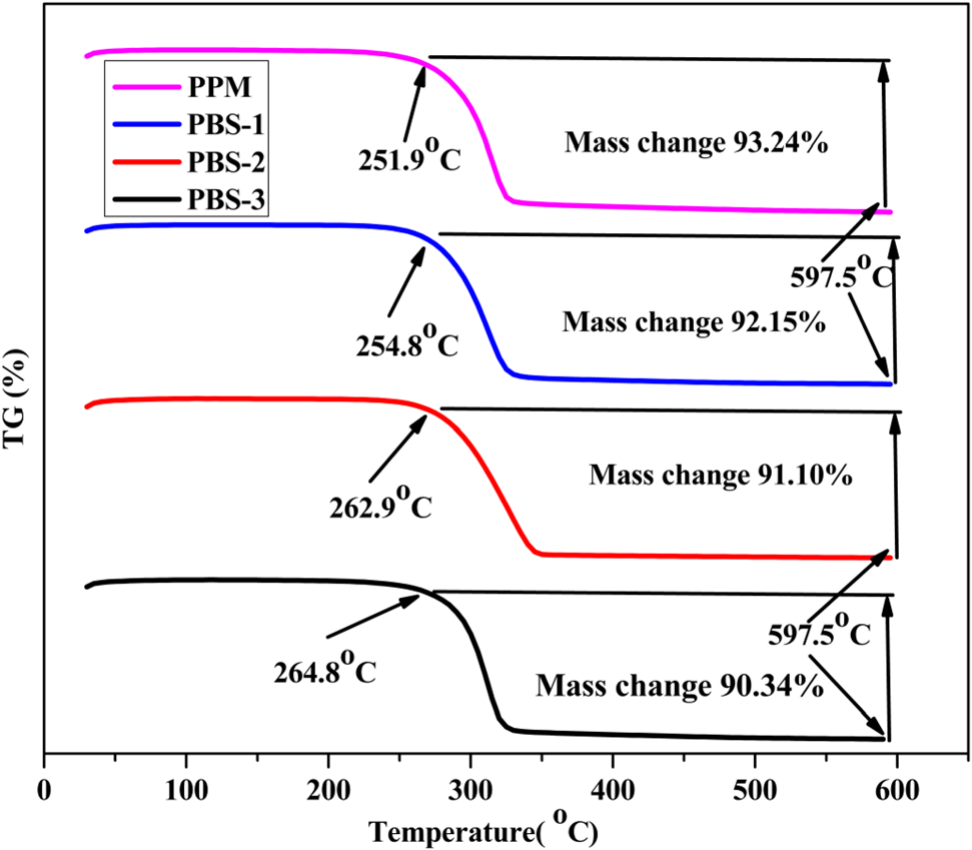
TG analysis of the PPM, PBS-1, PBS-2, and PBS-3 samples.

TGA testing plays a key role in understanding the thermal stability and degradation behavior of polymer-modified nanomaterials, which is an inherent test for assessing packaging compatibility. The TGA curves of the fabricated PLA-based nanomats revealed a one-stage degradative nature for all samples. PPM sample had an initial degradation temperature of 251.9 °C with a mass change of 93.24%. The onset degradation temperature showed a significant rise following the introduction of betel leaf extract and silvadur, leading to enhanced initial stability. PBS-1 was degraded at 254.8 °C with a mass loss of 92.15%, PBS-2 shifted to 262.9 °C with a mass loss of 91.10%, and PBS-3 displayed the highest initial degradation temperature at 264.8 °C with a mass loss of 90.34% shown in Fig. 12. The slow mass loss, abetted by the high char residue nature of the modified samples, is associated with the polyphenolic substances derived from the extract, which catalyze carbon-rich char at higher temperatures and provide a protective layer against thermal degradation. In addition, the silvadur adds thermally stable inorganic ash, enhancing stability. The initial degradation temperature for PBS-2 and PBS-3 compared with that of PPM was significantly higher (*p* < 0.05). Both PBS-2 and PBS-3 had higher thermal stability than pure PLA, indicating that they would suitable for antimicrobial packaging applications which require medium thermal resistance.^40^

#### 4.6.2 DSC analysis

**Fig. 13:**
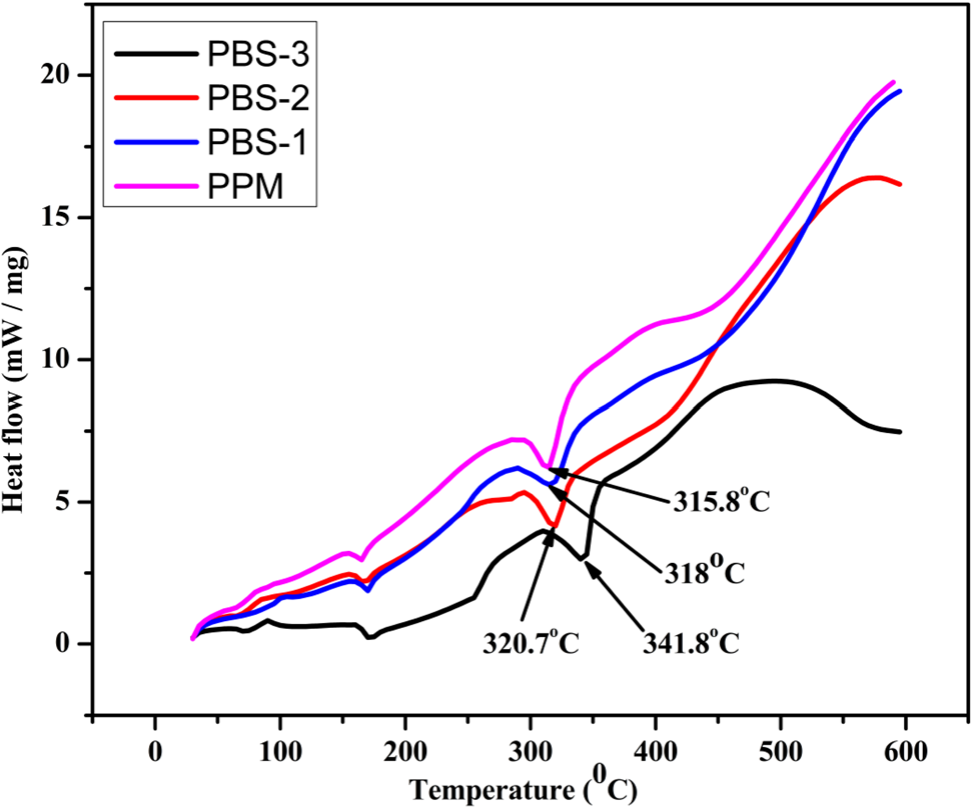
DSC analysis of PPM, PBS-1, PBS-2, and PBS-3 samples.

DSC is a thermoanalytical technique that most frequently records changes in heat-flow due to transitions of polymers, such as glass transition, crystallisation, melting and thermal degradation.^42^ DSC study for the thermal transitions related to nanomats is necessary in order to evaluate the chain mobility of the polymers, selectivity, and suitability for packaging use. It was observed from the DSC thermographs that for pure PLA (PPM), there is a decrease in transition temperature with values at 315.8 °C, which showed typical crystalline behavior toward pristine without stabilization adjuvants. Both using betel leaf extract and silvadur as additives, the transition temperatures apparently improved. PBS-1 displayed a Td value of approximately 318 °C, PBS-2 with 320.7 °C transition, and the highest value at 341.8 °C for PBS-3 reported in Fig. 13, which demonstrates an improvement in thermal properties with higher extract content. The positive shift in transition temperature is due to polyphenol compounds and inorganic fillers, which likely interact with PLA chains, limiting the movement of the macromolecules and therefore enhancing the thermal stability. These ingredients serve as thermal stabilizers, which reinforce the interactions between molecules by denser molecular packing. The highly uniform and continuous heat-flow behavior for all samples also testifies that the addition of extract and silvadur has not led to any structural disruption of PLA, but an improvement in its thermal behavior in a regulated manner.^40^ Altogether, Results showed that the transition temperature of PBS-3 was significantly higher than that for PPM, PBS-1 and PBS-2 (*p* < 0.05). Consequently, the DSC finding revealed that the modified nanomats, especially PBS-3 had better thermal transition behaviour and better application potential for food antimicrobial packaging.

#### 4.6.3 Derivative thermo-gravimetric analysis

**Fig. 14:**
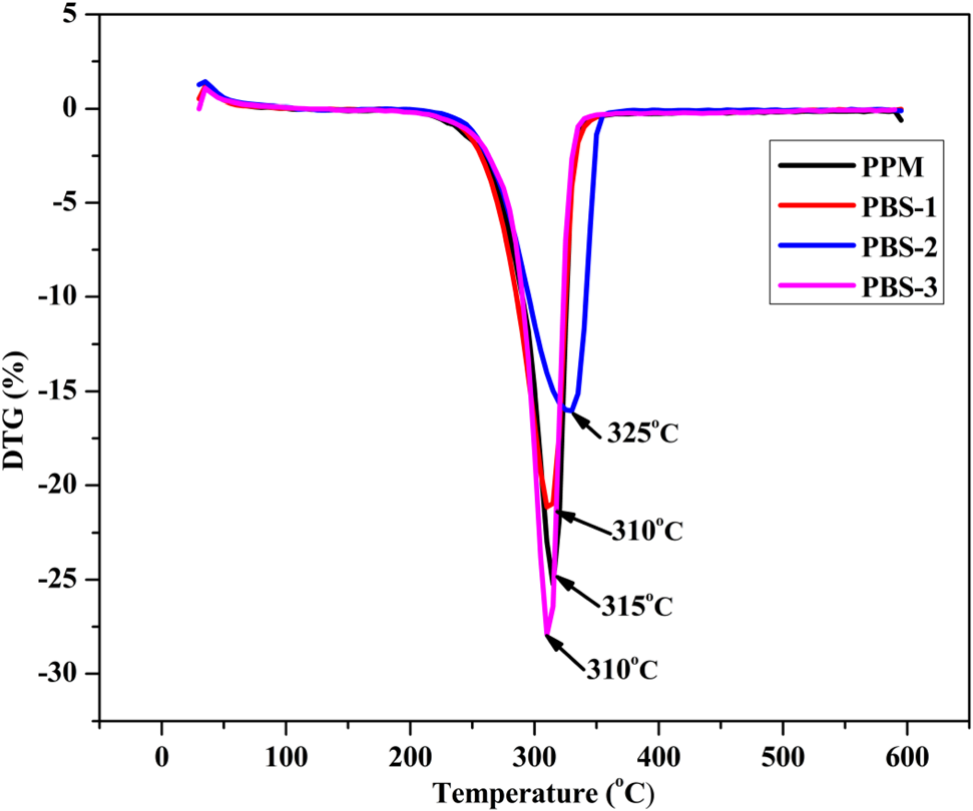
DTG analysis of PPM, PBS-1, PBS-2, and PBS-3 samples.

DTG is needed in order to determine the temperature at which the maximal rate of nanomats decomposition takes place, and this could explain a thermal breakdown behavior difference. There was only one main degradation peak in the DTG curves of all the samples. In the case of pure PLA (PPM), it showed its highest degradation rate at 315 °C, which is characteristic of decomposition behavior. After the modification, PBS-1 had a maximum at 310 °C, showing a slightly higher degradation rate. PBS-2, on the other hand, exhibited a maximum temperature at 325 °C, in support of high thermal stability (in terms of high decomposition peak temperature). PBS-3 also showed its maximum bio-deterioration rate back at 310 °C, like PBS-1, as highlighted in Fig. 14. Peak temperatures shifting for these peaks might be explained based on the combined effect of betel leaf extract and silvadur^40^ as follows: the extract consists of some thermolabile molecules that may lower Tmax in certain compositions (PBS-1, PBS-3), while a stronger interaction occurs between PLA and additional components in the case of PBS-2, causing a later time Tmax degradation. While these changes exist, all of them exhibit a one-step decomposition process demonstrating homogeneous degradative behavior among formulations.

### 4.7 Mechanical performance analysis

#### 4.7.1 Tensile strength analysis

**Fig. 15:**
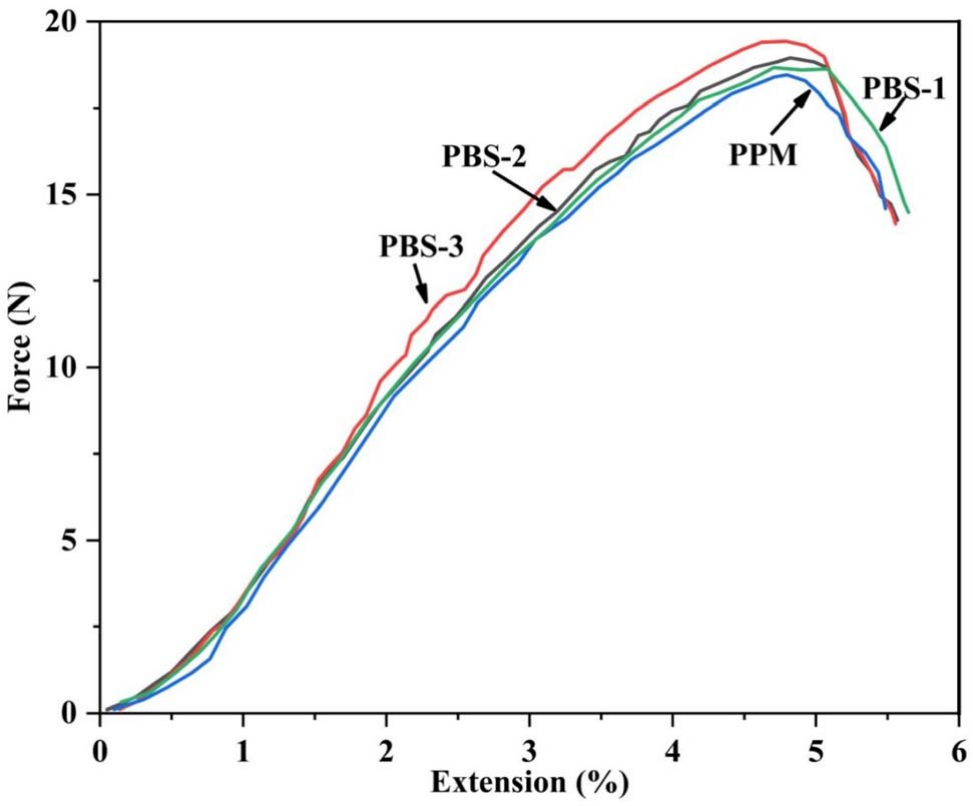
Tensile strength result comparison of PPM, PBS-1, PBS-2, and PBS-3 samples.

The test results shown in Fig. 15, indicate that the strength of the materials can be enhanced by the addition of silvadur and betel leaf extract to PLA. The force of the PPM samples was 18.52 N, that of PBS-1 being 18.96 N, indicating a little difference in strength and flexibility. PBS-2 showed a strength of 19.16 N, which was also increased to some extent. The PBS-3 showed the maximum strength of 19.41 N, which demonstrates that the level of extract contained in the nanomat endows both tensile strength and flexibility to composites. The enhancement of force is a result of betel leaf extract strengthening the PLA matrix, along with silvadur's stability from its antimicrobial characteristic. These adds increase the strength and flexibility of the material, and also provide a more suitable material for food packaging.^41^

#### 4.7.2 Bursting strength analysis

**Fig. 16:**
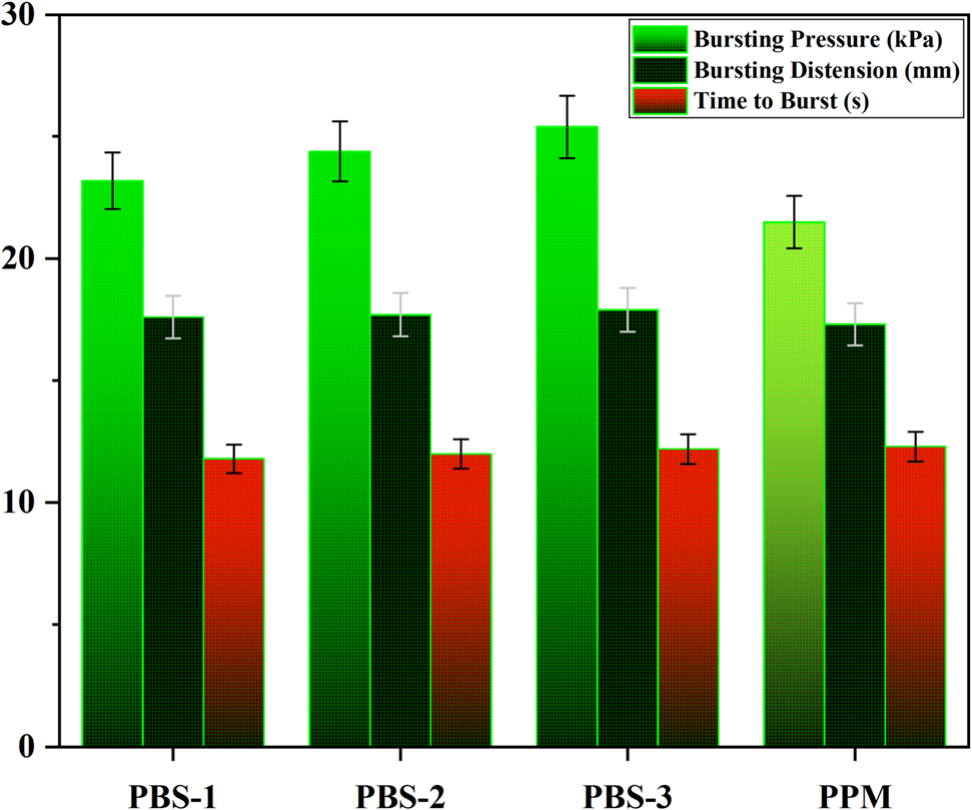
Bursting strength result comparison of PBS-1, PBS-2, PBS-3, and PPM samples.

As shown in Fig. 16, the bursting strength test was conducted to evaluate the ability of PLA-based nano mats to withstand multidirectional pressure, a vital mechanical property for antimicrobial food packages. The PPM sample showed a burst strength of about 21.5 kPa and a critical extension of ≈12.3 mm which was used as a mechanical reference point to compare the behavior of the new PLA nanofiber network being developed here. The bursting pressure with 11.8 mm of extension was significantly increased to 23.2 kPa for PBS-1, which may aid in stress distribution by introducing a low quantity of bioactive (acting as viscosity fillers) additives into the polymer matrix. The burst strength increased to 24.4 kPa with an elongation at break of 12 mm for PBS-2, showing more fiber–matrix interaction and improved densification of the nanofibrous structure. The maximum burst strength of PBS-3 (25.4 kPa, 12.2 mm) was significantly different compared to that of PPM (*p* < 0.05). This improvement can be due to synergistic interactions among PLA, betel leaf extract and silver-based additives. As a result, higher resistance against pressure along multidirectional was observed.

## 5 Conclusions

In this study, the silver nitrate and betel leaf extract incorporated with PLA-based electrospun nanomat for antimicrobial food-packaging applications. By electrospinning a PLA solution, uniform nanofibrous structures were obtained, and the functional characteristics of the PLA matrix could be enhanced by adding bioactive agents. Nanofiber formation and incorporation of the antibacterial agents was also confirmed through morphological, chemical, and elemental analyses. Due to its high moisture resistance, sufficient thermal stability and mechanical properties along with acceptable *in vitro* antibacterial and antifungal activity the developed nanomats can be used as food packaging materials. From all the formulations, PBS-3 exhibited an overall performance with relatively greater antimicrobial effectiveness, good barrier properties and better thermal and mechanical resistance than other samples. The long-term food storage performance, statistical validation for the structural and antimicrobial results, Ag^+^ ion migration, silver-resistant strains like *Escherichia coli* [pMG101], environmental impacts, regulatory compliance and process scalability would be the future scope of this study to facilitate the commercial application in sustainable packaging fields.

## Conflicts of interest

There are no conflicts to declare.

## Data Availability

The data supporting the findings of this study are available within the article.
